# Optic Disc and Cup Segmentation in Retinal Images for Glaucoma Diagnosis by Locally Statistical Active Contour Model with Structure Prior

**DOI:** 10.1155/2019/8973287

**Published:** 2019-11-20

**Authors:** Wei Zhou, Yugen Yi, Yuan Gao, Jiangyan Dai

**Affiliations:** ^1^College of Computer Science, Shenyang Aerospace University, Shenyang, Liaoning, China; ^2^School of Software, Jiangxi Normal University, Nanchang, Jiangxi, China; ^3^College of Information Science and Engineering, Northeastern University, Shenyang, Liaoning, China; ^4^School of Computer Engineering, Weifang University, Weifang, China

## Abstract

Accurate optic disc and optic cup segmentation plays an important role for diagnosing glaucoma. However, most existing segmentation approaches suffer from the following limitations. On the one hand, image devices or illumination variations always lead to intensity inhomogeneity in the fundus image. On the other hand, the spatial prior knowledge of optic disc and optic cup, e.g., the optic cup is always contained inside the optic disc region, is ignored. Therefore, the effectiveness of segmentation approaches is greatly reduced. Different from most previous approaches, we present a novel locally statistical active contour model with the structure prior (LSACM-SP) approach to jointly and robustly segment the optic disc and optic cup structures. First, some preprocessing techniques are used to automatically extract initial contour of object. Then, we introduce the locally statistical active contour model (LSACM) to optic disc and optic cup segmentation in the presence of intensity inhomogeneity. Finally, taking the specific morphology of optic disc and optic cup into consideration, a novel structure prior is proposed to guide the model to generate accurate segmentation results. Experimental results demonstrate the advantage and superiority of our approach on two publicly available databases, i.e., DRISHTI-GS and RIM-ONE r2, by comparing with some well-known algorithms.

## 1. Introduction

Glaucoma is the second leading cause of blinding in modern times and approximately 80 million persons to be afflicted with glaucoma by the year 2020 [1, 2]. Since the lost capabilities of the optic nerve fibers caused by glaucoma cannot be recovered, early detection and timely treatment of glaucoma can be regarded as the most effective way for patients to slow down the procession of visual damage.

**Figure 1:**
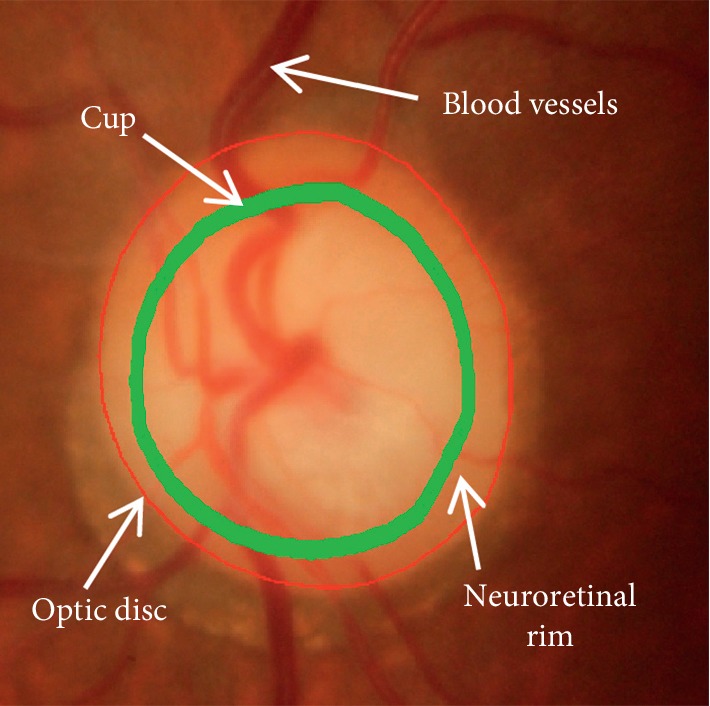
Major structures of the optic disc. Red line: the optic disc boundary; green line: the optic cup boundary; the region between the red line and green line is the neuroretinal rim.

Glaucoma screening is done by ophthalmologists who use the retinal fundus images to assess the damaged optic disc. Nevertheless, this process is subjective, time consuming, and expensive. Therefore, automatic glaucoma screening would be very beneficial [3]. There are two different regions within the optic disc. A center bright zone is the optic cup, and the peripheral region between the optic disc and optic cup boundaries is the neuroretinal rim (see the region of interest (ROI) depicted in Figure 1).

According to the characteristics of the optic disc, several strategies can be used to assess the optic disc. One of the effective strategies is to utilize the image features for optic disc assessment [4]. Nevertheless, how to select the suitable image features and classifiers is the challenging issue. Apart from the aforementioned feature extraction strategy, the usage of the clinical indicators is another strategy to assess the optic disc, such as the vertical cup to disc ratio (CDR) [5], ISNT rule [6], and notching. Although these clinical indicators are different from each other, precise optic disc and optic cup boundary information is necessary.

**Figure 2:**
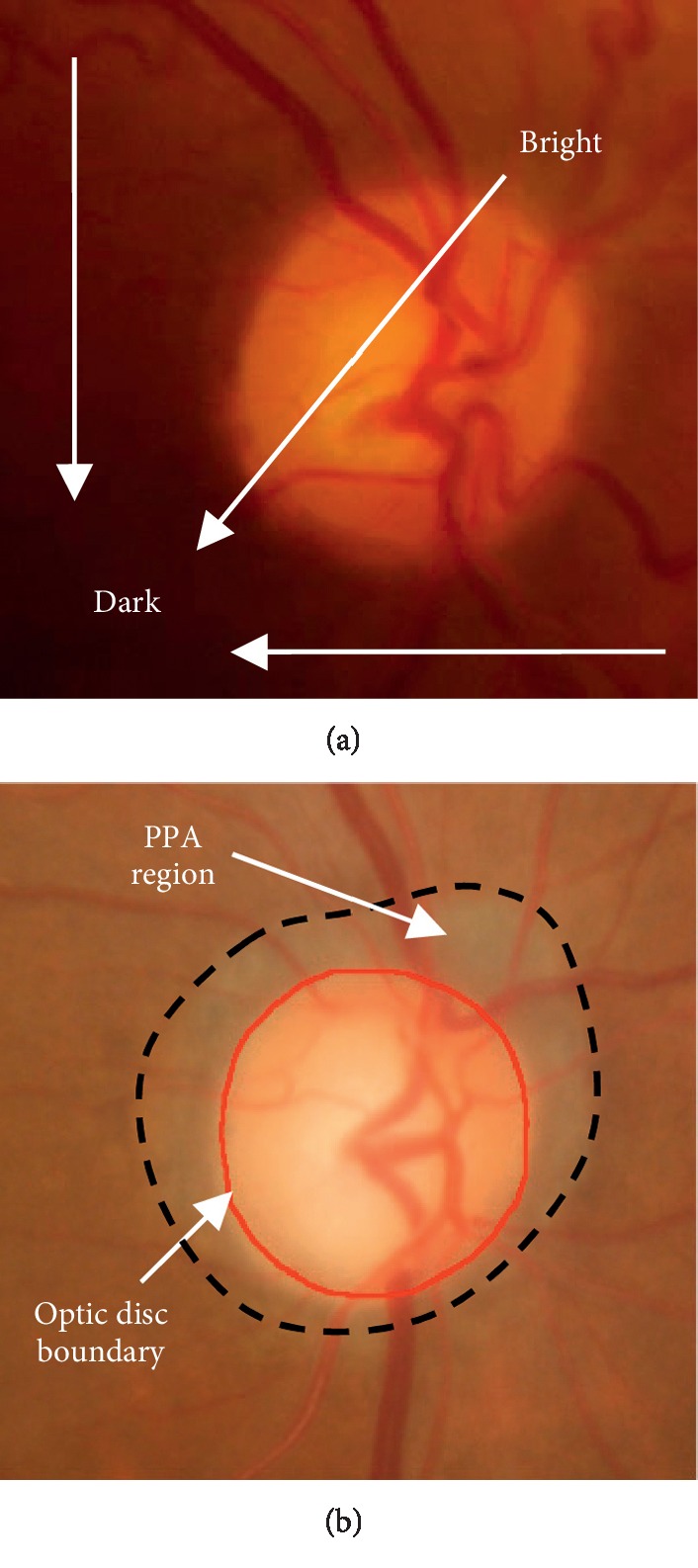
Intensity inhomogeneity challenges in optic disc and optic cup segmentation.

To date, there are a series of approaches that have been developed for optic disc and optic cup segmentation [7–27]. They can be roughly classified into the following two categories: nonmodel-based and model-based approaches [7]. For nonmodel-based approaches [3, 8–11], the contours of both the optic disc and the optic cup are extracted by means of some techniques, i.e., morphological operations, pixels clustering, and thresholding. However, intensity inhomogeneity is a frequently occurring phenomenon in retinal fundus images caused by imperfection of image devices or illumination variations, which affects the contour extraction of the optic disc (Figure 2(a)).

For model-based approaches, they can be divided into shape-based template-matching approaches [12–15], deformable model approaches [16–29], and deep learning-based model approaches [30–32]. Considering that the shape of the object is round or slightly oval, the contour of the optic disc can be estimated as a circle [12–14] or an ellipse [15]. However, the shape-based template-matching approaches cannot represent contours of complex topology and handle topological changes, such as the optic disc regions with shape irregularity due to some pathological changes (i.e., peripapillary atrophy (PPA), see Figure 2(b)) or variations in view.

To address it, many deformable model approaches have been presented which can be further divided into edge-based active contour models [16–21] and region-based active contour models [22–27]. Edge-based active contour models can bridge the discontinuities in the image feature being located. Besides, they can deform the shape of the object freely due to the fact that they have no global structure of the template. In [16, 17], the optic disc boundary is extracted by the gradient vector flow (GVF). Then, the optic disc contour is evolved via a minimization of the effect on the perturbance in the energy value due to the high variations at the vessels locations. In [18–21], the authors used a modified level set approach, with ellipse fitting to detect the optic disc and optic cup margins. Although these approaches perform well in most regular cases, an irregularly shaped optic disc having high gradient variations will fail in detecting the entire optic disc. More recently, motivated by the main idea of the Mumford-Shah model [28], some region-based active contour models such as Chan-Vese (C-V) model [22] and its variations [23–27] have been applied to optic disc contour extraction. Although these models can perform better in dealing with the local variations of the optic disc, they can hardly deal with the images with intensity inhomogeneity. In a recent paper, a robust level set approach called locally statistical active contour model (LSACM) is presented [29] by exploiting local image region statistics in an unsupervised manner. Compared with the existing region-based active contour models, LSACM approach performs better performance, especially for the image segmentation in the presence of intensity inhomogeneity. However, one difficulty with active contour model-based approaches is that the spatial correlation prior information between the optic disc and optic cup is ignored. Therefore, the useful spatial information cannot guide and constrain the contour evolution.

Nowadays, deep learning has widely been used in computer vision and pattern recognition areas and achieved remarkable performance, and some optic disc and optic cup segmentation approaches based on deep network have been proposed [30–32]. Although these approaches can achieve good performance in optic disc and optic cup segmentation, there are still some limitations in them [33, 34]. On the one hand, a large number of training samples consisting of pixel-level annotations are necessary to train the deep network model for testing. However, it is difficult for the network to achieve promising segmentation performance in the condition of insufficient labeled training samples. On the other hand, these networks ignore the prior knowledge of objects, thereby the spatial information being lost in encoder through max-pooling resulting in irregular segmentation.

**Figure 3:**
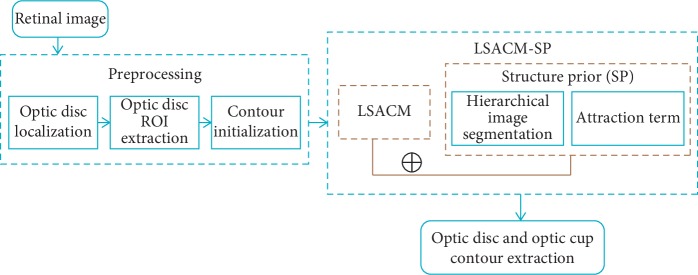
The flowchart of the proposed approach.

To address all the limitations raised above, a novel locally statistical active contour model with structure prior (LSACM-SP) approach is developed in this paper which aims at jointly segmenting optic disc and optic cup in retinal fundus images. The major innovations are as below: first of all, unsupervised LSACM is employed to join optic disc and optic cup segmentation over different retinal fundus images, which can address the issue of insufficient labeled samples and the influence of the retinal fundus images with a large range of appearance and illumination variations. Second, since the spatial correlation information is typically ignored by the existing deep network-based approaches, the segmentation performance is accordingly reduced. To address it, we design a structure prior to satisfy with the topological structure in retinal images that “cup” region is contained in the “disc” region. At last, we develop an efficient segmentation approach by incorporating locally statistical active contour model with the proposed structure prior together to simultaneously extract optic disc and optic cup contours. Since minimizing the proposed energy functional can acquire the contours, there is no need to use predefined geometric templates to guide auto-segmentation. Therefore, our approach reduces the segmentation performance degradation on the fundus images with a large range of intensity and shape variations. The overview of the proposed approach is shown in Figure 3.

The rest of this paper is organized as follows. First, some preprocessing techniques are depicted in Section 2. Then, the introduction of the proposed LSACM-SP is given in Section 3. Next, the experimental results and analyses are presented in Section 4. At last, we summarize this paper in Section 5.

## 2. Preprocessing

**Figure 4:**
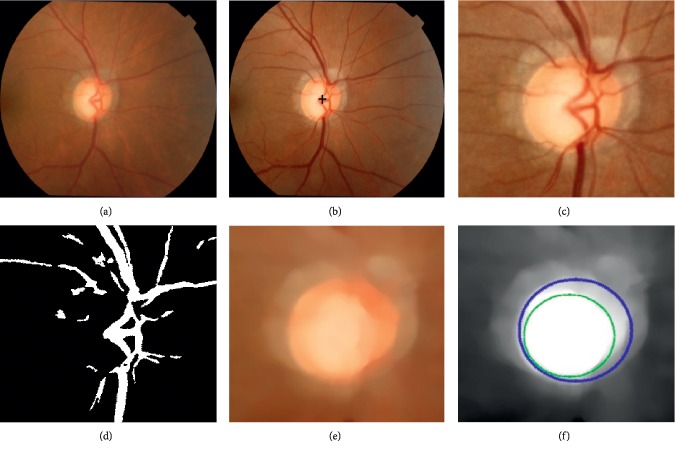
Preprocessing. (a) Original color image; (b) detected optic disc center in black “+”; (c) extracted ROI; (d) blood vessels extraction; (e) “vessels-free” image; (f) red channel of (e). Blue: the extracted optic disc initialization contour and green: the extracted optic cup initialization contour.

To perform accurate optic disc and optic cup segmentation, some preprocessing operations are necessary before carrying out our approach. In this paper, the preprocessing process consists of optic disc location, optic disc ROI extraction, and contour initialization.  Figure 4 depicts the process of preprocessing.

Given a retinal image (see Figure 4(a)), we employ our previous proposed approach based on saliency detection and feature extraction techniques to locate optic disc [1]. The detected optic disc location is marked in black “+” (see Figure 4(b)). Meanwhile, a 400 *∗* 400 ROI around the optic disc is extracted for further segmentation, as shown in Figure 4(c). Since blood vessels in the retinal images vary much in size, and meanwhile their locations and shapes among individual cases, a high variance in the data will affect the performance of segmentation (see Figure 4(c)). Hence, it is necessary to remove the influence caused by blood vessels before contour evolution. Recently, *B*-COSFIRE filters that combine the aligned responses of DoG filters with geometric mean are simple and robust for blood vessels segmentation. Therefore, we firstly use two rotation-invariant *B*-COSFIRE filters given in [35] to segment blood vessels, as illustrated in Figure 4(d). After obtaining the blood vessels, an image inpainting algorithm proposed in [36] is employed to fill in the blood vessel areas. In our setting, the blood vessel removal is to replace each vessel pixel intensity value by the median of the intensity values of the pixels in its neighborhood image that are not vessel pixels. According to our experience, we set the size of neighbor at 15. Meanwhile, image information around the vessel regions is used to fill in vessels and the obtained “vessels-free” image is shown in Figure 4(e). Considering that red color channel gives a good definition both on the optic disc and optic cup regions, this paper chooses the red channel of “vessels-free” image (see Figure 4(f)) for segmentation. For the obtained red channel of blood vessel-removed optic disc image, we use canny edge detection and circular Hough transform to estimate the positions and sizes of the optic disc and the optic cup [23]. In experiment, we set the parameters of threshold and sigma at 0.3 and 0.8, respectively, for canny edge detection. Finally, the estimated results are regarded as the initialization contours for our approach.

## 3. Methods

In Section 3.1, first of all, we briefly review the locally statistical active contour model (LSACM). Then, the structure prior is given in Section 3.2. Finally, we design a novel LSACM-SP model for simultaneous segmentation of optic disc and cup by joining the LSACM and the structure prior together in Section 3.3. In order to better understand the proposed approach, in the following sections, we will use bold italic variables (e.g., **x**, **y**) to denote vectors, small letters (e.g., *n*) to denote scalars, and capital letters (e.g., *I*) to denote functions.

### 3.1. Locally Statistical Active Contour Model (LSACM)

According to [37], we can learn that the regions in the images with severe intensity inhomogeneity must have sharp discontinuities in the statistics. Inspired by it, Zhang et al. [29] presented a locally statistical active contour model (LSACM) to deal with the images with intensity inhomogeneity. In [29], the authors firstly modeled the objects with intensity inhomogeneity as Gaussian distributions of different means and variances. Then, transforming the pixels in original image into another domain makes the intensities in the transformed space having less overlapping in the statistics. Besides, to approximate the true image, a maximum likelihood energy functional is employed. In comparison with the existing statistical model-based segmentation algorithms [23–28], LSACM is more robust and stable. For more detailed descriptions, refer to [29].

For a given input image *I*, its segmentation can be done by minimizing the following energy functional:

(1) $$\begin{array}{c} E^{\text{LSACM}}\left(\theta ,B,\Phi \right)=\overset{n}{\underset{i=1}{\sum }}\int_{\Omega }F_{i}M_{i}\left(\Phi \left(y\right)\right)\text{d}y, \end{array}$$

where

(2) $$\begin{array}{c} F_{i}≜\int_{\Omega }K_{\rho }\left(x,y\right)\left(\log\left(\sigma_{i}\right)+\left(\frac{{\left(I\left(y\right)-B\left(x\right)c_{i}\right)}^{2}}{2\sigma_{i}^{2}}\right)\right)\text{d}x,\\ K_{\rho }\left(x,y\right)=\left\{\begin{array}{cc} 1, & \left|y-x\right|\leq \rho ,\\ 0, & \text{else}, \end{array}\right.\\ M_{i}\left(\Phi \left(y\right)\right)=\left\{\begin{array}{cc} 1, & y\in \Omega_{i},\\ 0, & \text{else,} \end{array}\right. \end{array}$$

where *n* is the number of objects in *I*. **x**, **y** ∈ *Ω* ⊂ *ℜ*^2^ are pixel coordinates; *Ω*=∪_*i*=1,…,*n*_*Ω*_*i*_ represents image domain, in which *Ω*_*i*_ is the domain of the *i*-th object and *Ω*_*i*_∩*Ω*_*j*_=Θ for all *i* ≠ *j*. The symbol Θ denotes an empty set. *I*(**y**) is the pixel value at the location of pixel coordinate *y*. *B*(**x**) : *Ω*⟶*ℜ* is an unknown bias field. Φ is a series of level set functions. If **y** ∈ *Ω*_*i*_, then *M*_*i*_(Φ(**y**))=1, otherwise *M*_*i*_(Φ(**y**))=0 in which *M*_*i*_(Φ(**y**)) is the membership function of the region *Ω*_*i*_. The symbol *O*_*x*_ denotes a neighboring region centering at location **x**, i.e., *O*_*x*_={**y**||**y** − **x**| ≤ *ρ*}, **y** is the neighborhood point relative to **x**, and *ρ* is the radius of the region *O*_*x*_. The symbol ***θ***={*c*_*i*_, *σ*_*i*_, *i*=1,2,…, *n*} are needed to be estimated parameters, where the constant *c*_*i*_ is the true signal of the *i*-th object and the object in region *Ω*_*i*_ is assumed to be Gaussian distributed with standard deviation *σ*_*i*_. *B*(**x**)*c*_*i*_ is the spatial varying mean that is estimated at the local region *Ω*_*i*_∩*O*_*x*_. *K*_*ρ*_(**x**, **y**) denotes the indicator function.

**Figure 5:**
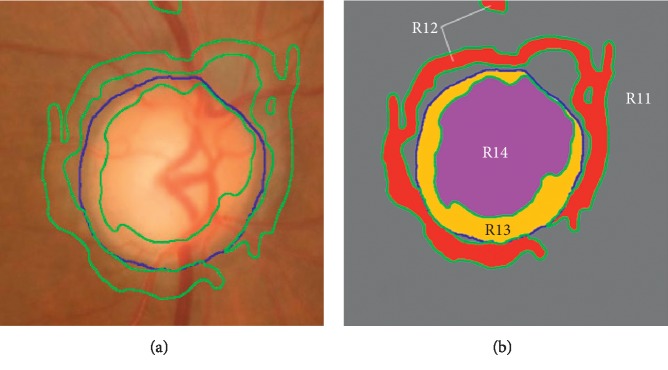
Segmentation results obtained by LSACM. (a) Segmentation results by the four-phase-based LSACM and (b) the corresponding regions by four-phase LSACM. Green: optic cup result and blue: optic disc result.

Considering that one level set function Φ just represents two different regions, namely, the inside region of contour *S*, *Ω*_1_=in(*S*)={Φ > 0} and the outside region of contour *S*, *Ω*_2_=out(*S*)={Φ < 0}, this model is defined as two phase. However, one level set cannot work well for more than two different regions. To deal with this case, incorporating four-phase model is necessary, which uses two level set functions, i.e., Φ_1_ and Φ_2_, to represent all different regions [29]. Hence, four-phase model denotes any two adjacent regions indicated by different colors.  Figure 5(a) depicts the segmentation results obtained from the four-phase LSACM, in which the blue line and the green line represent two-level set functions (i.e., Φ_1_ and Φ_2_), respectively. The corresponding regions, such as “R11” (in gray color), “R12” (in red color), “R13” (in yellow color), and “R14” (in purple color) depicted in Figure 5(b), are extracted from Figure 5(a). Seen from the segmentation results in Figure 5(b), optic disc and optic cup regions cannot be determined directly due to the existing nonobject segmentation region (i.e., “R12”). The main reason is that the evolution processes of the regions from different classes (i.e., “R11,” “R12,” “R13” and “R14”) are independent while ignoring the prior that “cup” region is contained in the “disc” region. Therefore, how to control the evolution of two level set functions which one locates inside another, to approximate the true optic disc and optic cup contours, is a main issue.

### 3.2. Structure Prior

To address the aforementioned issue, this paper proposes a structure prior to guarantee the truth that the “cup” region is contained in the “disc.” The structure prior together with the effective optimization of LSACM can enable the proposed approach generate robust and reliable segmentation results. In this paper, the structure prior consists of hierarchical image segmentation and attraction term. Specifically, hierarchical indicates evolution manner of two-level set functions, that is, the evolution of optic disc is performed on the whole image region while the evolution of optic cup is constrained inside the optic disc region. The symbols *ϕ* and *φ* are two-level set functions. In this study, we use *ϕ* > 0 and *φ* > 0 to represent the optic disc region and the optic cup region, respectively. The whole image region represented as *Ω* and *E* is an energy functional.

#### 3.2.1. Hierarchical Image Segmentation

Observing that the optic cup in the retinal image always places inside the optic disc, optic cup segmentation is enough to be done inside the optic disc. Here, we propose the hierarchical image segmentation *E*^Cup^(*c*_3_, *c*_4_, *σ*_3_, *σ*_4_, *B*_cup_(**x**), *ϕ*, *φ*) to indicate the image region in which the contour evolution of optic cup is enforced. We consider

(3) $$\begin{array}{c} E^{\text{Cup}}\left(c_{3},c_{4},\sigma_{3},\sigma_{4},B_{\text{cup}}\left(x\right),\phi ,\varphi \right)=E^{H}\left(c_{3},c_{4},\sigma_{3},\sigma_{4},B_{\text{cup}}\left(x\right),\phi ,\varphi \right)+E^{\text{HCSmooth}}\left(\varphi \right), \end{array}$$

(4) $$\begin{array}{c} E^{H}\left(c_{3},c_{4},\sigma_{3},\sigma_{4},B_{\text{cup}}\left(x\right),\phi ,\varphi \right)=\alpha \left\{\int_{\phi >0}\int_{\Omega }K_{\rho }\left(x,y\right)\left(\log\left(\sigma_{3}\right)\right)+\left(\frac{{\left(I\left(y\right)-B_{\text{Cup}}\left(x\right)c_{3}\right)}^{2}}{2\sigma_{3}^{2}}\right)H\left(\varphi \left(y\right)\right)\text{d}x\text{ d}y+\int_{\phi >0}\int_{\Omega }K_{\rho }\left(x,y\right)\left(\left(\log\left(\sigma_{4}\right)\right)+\left(\frac{{\left(I\left(y\right)-B_{\text{Cup}}\left(x\right)c_{4}\right)}^{2}}{2\sigma_{4}^{2}}\right)\right)\left(1-H\left(\varphi \left(y\right)\right)\right)\text{d}x\text{ d}y\right\}, \end{array}$$

(5) $$\begin{array}{c} E^{\text{HCSmooth}}\left(\varphi \right)=\mu \int_{\Omega }\left|\nabla H\left(\varphi \left(y\right)\right)\right|dy, \end{array}$$

where *E*^*H*^(*c*_3_, *c*_4_, *σ*_3_, *σ*_4_, *B*_cup_(**x**), *ϕ*, *φ*) is used to constrain the evolution of the optic cup which should be done inside the optic disc region. Thus, the background region is not considered in (3) while reducing the influence of nonobjects. *E*^HCSmooth^(*φ*) is used to make the extracted contour more smoother. *B*_Cup_ is an unknown bias field for segmenting the OC, which accounts for the intensity inhomogeneity in the optic disc. *α* and *μ* are positive parameters and the level set functions *ϕ* and *φ* are positive inside zero-level sets. *H* represents the Heaviside function. ∇ is the gradient operator. *σ*_3_ is the standard deviation subject to the object in the optic cup region and *σ*_4_ is the standard deviation subject to the object in the neuroretinal rim region.

#### 3.2.2. Attraction Term

The optic cup region *φ* > 0 should be in the optic disc region *ϕ* > 0 to reduce the background region. A term measuring the intersection area of the regions {*ϕ* < 0} and {*φ* > 0} plays a role to pull the region {*φ* > 0} to inside the region {*ϕ* > 0}:

(6) $$\begin{array}{c} E^{\text{Attraction}}\left(\phi ,\varphi \right)=\nu \int_{\Omega }\left(1-H\left(\phi \left(y\right)\right)\right)H\left(\varphi \left(y\right)\right)\text{d}y, \end{array}$$

where *ν* is a positive parameter. Obviously, the objective function value in (6) becomes zero when the region *φ* > 0 is inside the region *ϕ* > 0.

### 3.3. LSACM-SP

**Figure 6:**
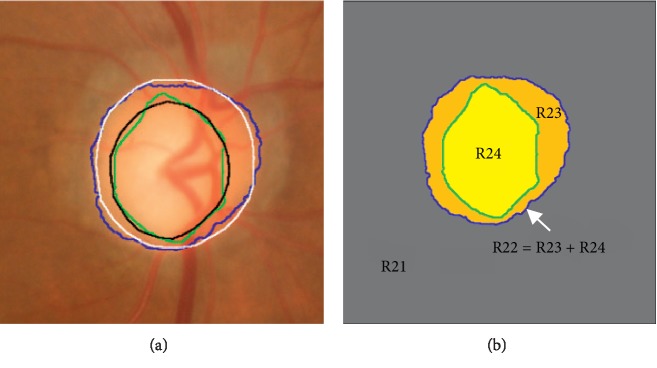
The segmentation results by the proposed model. (a) Segmentation results by the proposed LSACM-SP and (b) the corresponding regions by the proposed LSACM-SP. White: optic disc ground truth by an expert, black: optic cup ground truth by an expert, green: optic cup result by an approach, and blue: optic disc result by an approach.

The proposed model is designed by combining the locally statistical model and structure prior (hierarchical image segmentation and attraction term) together. The details of the proposed LSACM-SP model are given as follows:

(7) $$\begin{array}{c} E^{\text{LSACM}-\text{SP}}\left(\theta ,B,\phi ,\varphi \right)=E^{\text{Disc}}\left(c_{1},c_{2},\sigma_{1},\sigma_{2},B_{\text{Disc}}\left(x\right),\phi \right)+E^{\text{Cup}}\left(c_{3},c_{4},\sigma_{3},\sigma_{4},B_{\text{cup}}\left(x\right),\phi ,\varphi \right)+E^{\text{Attraction}}\left(\phi ,\varphi \right), \end{array}$$

where

(8) $$\begin{array}{c} E^{\text{Disc}}\left(c_{1},c_{2},\sigma_{1},\sigma_{2},B_{\text{Disc}}\left(x\right),\phi \right)=E^{\text{LSACM}}\left(c_{1},c_{2},\sigma_{1},\sigma_{2},B_{\text{Disc}}\left(x\right),\phi \right)+E^{\text{DSmooth}}\left(\phi \right), \end{array}$$

(9) $$\begin{array}{c} E^{\text{LSACM}}\left(c_{1},c_{2},\sigma_{1},\sigma_{2},B_{\text{Disc}}\left(x\right),\phi \right)=\int_{\Omega }\int_{\Omega }K_{\rho }\left(x,y\right)\left(\log\left(\sigma_{1}\right)+\left(\frac{{\left(I\left(y\right)-B_{\text{Disc}}\left(x\right)c_{1}\right)}^{2}}{2\sigma_{1}^{2}}\right)\right)H\left(\phi \left(y\right)\right)\text{d}x\text{ d}y+\int_{\Omega }\int_{\Omega }K_{\rho }\left(x,y\right)\left(\log\left(\sigma_{2}\right)+\left(\left(\frac{{\left(I\left(y\right)-B_{\text{Disc}}\left(x\right)c_{2}\right)}^{2}}{2\sigma_{2}^{2}}\right)\right)\right)\left(1-H\left(\phi \left(y\right)\right)\right)\text{d}x\text{ d}y, \end{array}$$

(10) $$\begin{array}{c} E^{\text{DSmooth}}\left(\phi \right)=\lambda \int_{\Omega }\left|\nabla H\left(\phi \left(y\right)\right)\right|\text{d}y, \end{array}$$

where ***θ***={*c*_*i*_, *σ*_*i*_, *i*=1,2,3,4}, *B*={*B*_Disc_(**x**), *B*_Cup_(**x**)}, and *E*^LSACM^(*c*_1_, *c*_2_, *σ*_1_, *σ*_2_, *B*_Disc_, *ϕ*) and *E*^DSmooth^(*ϕ*) are the original LSACM model and the smoothing term, respectively. *B*_Disc_(**x**) is an unknown bias field for segmenting the optic disc. *λ* is a positive parameter. *σ*_1_ is the standard deviation subject to the object in the optic disc region and *σ*_2_ is the standard deviation subject to the object in the background region (the outside region of the optic disc). The segmentation result obtained by LSACM-SP is illustrated in Figure 6(a), and the corresponding segmentation regions are shown in Figure 6(b). Here, “R24” is the optic cup region, “R23” is the neuroretinal rim region, “R22” is the optic disc region consisting of “R23” and “R24,” and “R21” is the background region.

In the proposed segmentation approach, we set *n* as 4 indicating 4 regions, namely, outer and inner regions of the optic disc (“R21” and “R22” in Figure 6(b)) and the outer and inner regions of the optic cup (“R23” and “R24” in Figure 6(b)). Specially, the outer region of optic cup (“R23”in Figure 6(b)) is the complementary set of the inner optic cup region (“R24” in Figure 6(b)) relative to the optic disc region (“R22” in Figure 6(b)). According to the aforementioned descriptions, we further rewrite (7) and obtain the objective function as

(11) $$\begin{array}{c} E^{\text{LSACM}-\text{SP}}\left(\theta ,B,\;\phi ,\varphi \right)=\overset{2}{\underset{i=1}{\sum }}\int_{\Omega }F_{i}M_{\text{Disc}\_i}\text{d}y\;+\lambda \int_{\Omega }\left|\nabla H\left(\phi \left(y\right)\right)\right|\text{d}y+\alpha \overset{4}{\underset{i=3}{\sum }}\int_{\Omega }F_{i}M_{\text{cup}\_i}\text{d}y\;+\mu \int_{\Omega }\left|\nabla H\left(\varphi \left(y\right)\right)\right|\text{d}y+\nu \int_{\Omega }\left(1-H\left(\phi \left(y\right)\right)\right)H\left(\varphi \left(y\right)\right)\text{d}y, \end{array}$$

where

(12) $$\begin{array}{c} F_{1}\left(c_{1},\sigma_{1},B_{\text{Disc}}\left(x\right)\right)=\int_{\Omega }K_{\rho }\left(x,y\right)\left(\log\left(\sigma_{1}\right)+\left(\frac{{\left(I\left(y\right)-B_{\text{Disc}}\left(x\right)c_{1}\right)}^{2}}{2\sigma_{1}^{2}}\right)\right)\text{d}x,\\ F_{2}\left(c_{2},\sigma_{2},B_{\text{Disc}}\left(x\right)\right)=\int_{\Omega }K_{\rho }\left(x,y\right)\left(\log\left(\sigma_{2}\right)+\left(\frac{{\left(I\left(y\right)-B_{\text{Disc}}\left(x\right)c_{2}\right)}^{2}}{2\sigma_{2}^{2}}\right)\right)\text{d}x,\\ F_{3}\left(c_{3},\sigma_{3},B_{\text{cup}}\left(x\right)\right)=\int_{\Omega }K_{\rho }\left(x,y\right)\left(\log\left(\sigma_{3}\right)+\left(\frac{{\left(I\left(y\right)-B_{\text{cup}}\left(x\right)c_{3}\right)}^{2}}{2\sigma_{3}^{2}}\right)\right)\text{d}x,\\ F_{4}\left(c_{4},\sigma_{4},B_{\text{cup}}\left(x\right)\right)=\int_{\Omega }K_{\rho }\left(x,y\right)\left(\log\left(\sigma_{4}\right)+\left(\frac{{\left(I\left(y\right)-B_{\text{cup}}\left(x\right)c_{4}\right)}^{2}}{2\sigma_{4}^{2}}\right)\right)\text{d}x, \end{array}$$

(13) $$\begin{array}{c} M_{\text{Disc}\_1}\left(\phi \right)=H\left(\phi \left(y\right)\right),\\ M_{\text{Disc}\_2}\left(\phi \right)=1-H\left(\phi \left(y\right)\right),\\ M_{\text{Cup}\_3}\left(\phi ,\varphi \right)=H\left(\phi \left(y\right)\right)\left(H\left(\phi \left(y\right)\right)\right),\\ M_{\text{Cup}\_4}\left(\phi ,\varphi \right)=H\left(\phi \left(y\right)\right)\left(1-H\left(\phi \left(y\right)\right)\right), \end{array}$$

where ***θ***={*c*_*i*_, *σ*_*i*_, *i*=1,2,3,4}, **B**={*B*_Disc_(**x**), *B*_Cup_(**x**)}, *M*_Disc_1_ and *M*_Disc_2_ denote the membership functions for inner and outer regions of the optic disc, and *M*_Cup_3_ and *M*_Cup_4_ are the membership functions for inner and outer regions of the optic cup in the optic disc. Although the membership functions in (13) are alike with the four-phase model in LSACM, they are different in fact. The main difference is that the proposed approach incorporates the structure prior knowledge to constrain the contour evolution based on hierarchical. Therefore, the evolution space of feasible segmentation is reduced, which improves the accuracy of segmentation.

Seen from (7), it requires a hard work to find a minimizer *E*^LSACM−SP^ for *ϕ*, *φ*, ***θ***={*c*_*i*_, *σ*_*i*_, *i*=1,2,3,4}, and **B**={*B*_Disc_(**x**), *B*_Cup_(**x**)}, simultaneously. Similar to the [29], we solve the minimization problem for each variable alternatively to find a minimizer of (7). The procedure will be repeated until satisfying a stopping condition. In this study, the initial conditions of the gradient descent method are given as *B*_Disc_(**x**)=1, *B*_Cup_(**x**)=1, *σ*_*i*_=*i*(*i*=1,2,3,4), and the region scale parameter *ρ* is set as 6. Accordingly, the initialization of *c*_*i*_(*i*=1,2,3,4) can be calculated by (A.2)–(A.5). Meanwhile, the time step for level set evolution is set at Δ*t*_1_=1 and the time step regularization is set at Δ*t*=1. The level set functions *ϕ*=*ϕ*_0_ and *φ*=*φ*_0_. For more details, refer to Appendix.

## 4. Results

### 4.1. Database

In this paper, two publicly available databases, namely, DRISHTI-GS [38] and RIM-ONE r2 [39], are used to verify the effectiveness of our approach.

The DRISHTI-GS database [38] contains a total of 101 images containing 31 normal images and 70 glaucomatous images in 2896 × 1944 resolutions. For each image, the optic disc and optic cup are accurately annotated via a majority voting manual markings obtained from four glaucoma ophthalmologists. In our experiments, we use the marking result obtained by a value of threshold 0.75 as the final ground truth for evaluation.

The RIM-ONE r2 database [39] contains 455 retinal fundus images with 255 normal images and 200 glaucoma images. In experiment, all of the images in the database are firstly arranged in the same dimension by resizing, and then the preprocessing techniques depicted in Section 2 are used to these images. Finally, the obtained red channel of blood vessel-removed optic disc image and extracted initialization contours are regarded as the inputs for our approach.

**Figure 7:**
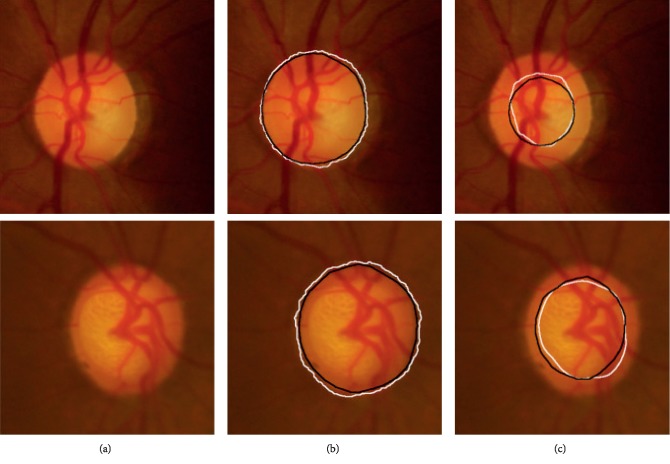
Optic disc and optic cup boundaries extraction. (a) Original color images, (b) original color images with contours of both optic disc and ground truth superimposed, and (c) original color images with contours of both optic cup and ground truth superimposed. Black line: ground truth marked by an expert and white line: results obtained by the proposed method.

In this paper, all the experiments are evaluated under the Matlab programming environment and on a desktop of 3.30 GHz CPU with 16G RAM.  Figure 7 illustrates two examples for the application of our model to optic disc and optic cup segmentation, in which Figure 7(a) gives the original image; Figure 7(b) shows the optic disc and the corresponding ground truth image; and Figure 7(c) depicts the optic cup and the corresponding ground truth.

### 4.2. Evaluation Measurements

In our experiment, three widely used measurements are utilized to evaluate the performances of different approaches, including Dice coefficients (DI), boundary-based distance, and accuracy.

Dice coefficients (DI):

(14) $$\begin{array}{c} \text{DI}=\frac{2\times N_{\text{TP}}}{2\times N_{\text{TP}}+N_{\text{FP}}+N_{\text{FN}}}, \end{array}$$

where *N*_TP_ is the number of true positive, *N*_FP_ is the number of false positive, and *N*_FN_ is the number of false negative. Positive and negative refer to pixels belonging to the segmentation area and background in accordance to the ground truth segmentation, respectively. The DI is a standard evaluation metric for segmentation tasks [18].

**Figure 8:**
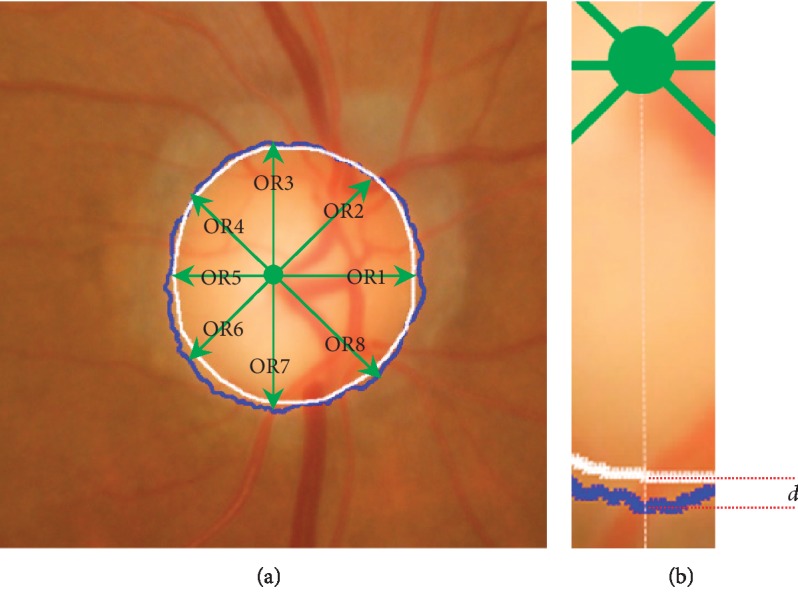
The description of boundary-based distance measurement. (a) The reference point (expert's curve centroid) and eight directions (OR1-OR8); (b) the detail of the distance between the expert's curve *C*_*g*_ (white) and our method's curve *C*_*o*_ (blue).

Boundary-based distance:

(15) $$\begin{array}{c} D=\frac{1}{k}\overset{\theta_{k}}{\underset{\theta =1}{\sum }}\left|d_{g}^{\theta }-d_{o}^{\theta }\right|, \end{array}$$

where *d*_*g*_^*θ*^ and *d*_*o*_^*θ*^ represent the distances from the expert's curve centroid to the points on the expert's curve *C*_*g*_ and our method's curve *C*_*o*_, respectively, in the *θ*-th angular direction and *k* denotes the count of angular samples. According to [23], we set *k* as 8 along angular directions 0°, 45°, 90°, 135°, 180°, 225°, 270°, and 315°, respectively. Ideally, the distance *D* should be close to zero.  Figure 8 gives a detailed description of boundary-based distance.  Figure 8(a) shows the center of expert's curve and eight directions (OR1–OR8).  Figure 8(b) depicts the distance between *C*_*g*_ and *C*_*o*_.

Accuracy:

(16) $$\begin{array}{c} \text{Acc}=\frac{\text{TP}+\text{TN}}{\text{TP}+\text{TN}+\text{FP}+\text{FN}}, \end{array}$$

where true positive (TP) is the number of glaucoma images that are correctly identified, false negative (FN) is the number of incorrectly found as nonglaucoma images, false positive (FP) is the number of incorrectly found as glaucoma images, and true negative (TN) is the number of nonglaucoma images that are correctly identified. Positive and negative refer to testing retinal images belonging to glaucoma and normal in accordance to the vertical CDR value. Similar to the existing approaches [3], when vertical CDR value is greater than a threshold, it is glaucomatous, otherwise, healthy, we set the standard threshold value as 0.5 for glaucoma diagnosis in this paper [40].

### 4.3. Optic Disc Segmentation Results

**Figure 9:**
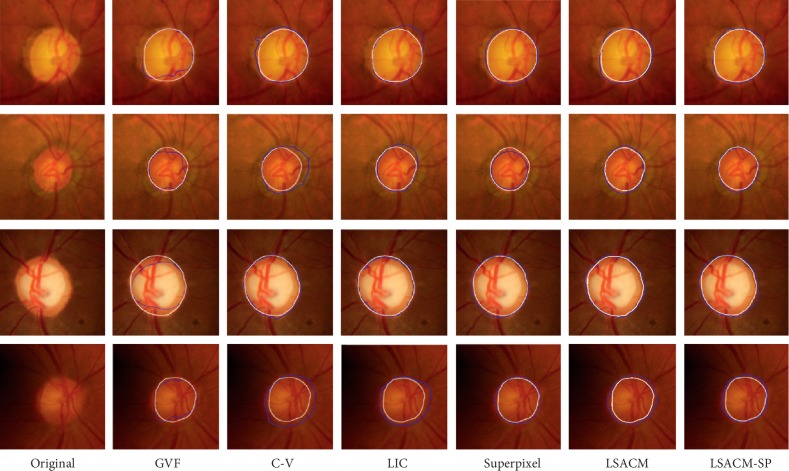
Optic disc segmentation results. First column: original images; second column: GVF model results [16]; third column: C-V model results [24]; fourth column: LIC model results [41]; fifth column: superpixel [3]; sixth column: LSACM [29]; seventh column: LSACM-SP. White line: ground truth and blue line: detected result by an approach.

In this section, five models are implemented for optic disc segmentation performance comparison, namely, gradient vector flow (GVF) [16], C-V model [24], LIC model [41], superpixel [3], and LSACM [29]. Several optic disc segmentation results obtained by different approaches are shown in Figure 9. As shown in this figure, the white lines show the segmentation results marked by the expert and the blue one by an approach. Since the first two row images in Figure 9 contain PPA having high gradient variations, all the segmentation approaches have error segmentation results, but our approach, LSACM approach, and superpixel approach are better than the others. For an irregular shaped optic disc image example with high gradient variations depicted in the fourth row, the segmentation results by the GVF model are sensitive to the local gradient minima. Although the C-V model can deal with the local gradient variations, it is not suitable to deal with intensity inhomogeneity image due to the fact that it uses piecewise constant functions to model images. For the LIC model based on locally weighted *K*-means clustering approach, it may fail to discriminate the intensities of an object from its background when the intensity inhomogeneity is severe. The main reason is that the clustering variance is ignored in it. Although superpixel-based approach can improve the segmentation performance by extracting features from the superpixel level, it may have a bias of underestimating large optic discs and overestimating small optic discs when the medium-sized optic discs are employed to train the model. Moreover, LSACM and our approaches are robust to intensity inhomogeneity (i.e., PPA). However, our approach takes the spatial correlation prior information into consideration while LSACM does not. Therefore, our approach is more robust than other comparison approaches.

To assess the performance of our approach, we compare it with other approaches in terms of DI and boundary-based distance measurements, as shown in Table 1. Seen from Table 1, our approach can achieve the highest DI and lowest average boundary distance among all the approaches, indicating the effectiveness of the proposed approach.

Seen from the aforementioned comparison results, our approach performs the best. The main reason is that the proposed model takes local image region statistics information into consideration, which is robust to noise and intensity overlapping (i.e., PPA). Besides, based on the incorporation of structure prior, all of the nonobject regions outside the optic disc are regarded as the background region in the proposed approach (see “R21” in Figure 6(b)), and thus all nonobject segmentation regions are eliminated (see “R12” in Figure 5(b)). Finally, an optic disc with fuzzy boundary is depicted in the fourth row. Comparing the results of our approach with those of the existing approaches, the optic disc boundary obtained by our approach is matching closely with the ground truth. The reason is that our approach models the objects as Gaussian distributions with different means and variances. Therefore, different objects will be separated from each other. Overall, the proposed optic disc segmentation approach is robust to a large range of variations in retinal images.

In order to further verify the effectiveness of the proposed LSACM-SP, the pairwise one-tailed *t*-tests is used in this paper. In this test, the null hypothesis is our LSACM-SP makes no difference when compared with the existing optic disc segmentation approaches and the alternative hypothesis is our LSACM-SP makes an improvement when compared with other approaches. For example, if we want to compare the performance of LSACM-SP with that of C-V (LSACM-SP vs. C-V), the null and alternative hypotheses are defined as *H*_0_ : *M*_LSACM‐SP_=*M*_C‐V_ and *H*_1_ : *M*_LSACM‐SP_ > *M*_C‐V_, respectively, where *M*_LSACM‐SP_ and *M*_C−V_ are the measurement results obtained by LSACM-SP and C-V approaches on different datasets. In our experiment, we set the significance level at 0.05. Seen from Table 2, all the *p* values are much less than 0.05, which means that the null hypotheses are disapproved in all pairwise tests. Therefore, the proposed LSACM-SP significantly outperforms other optic disc segmentation approaches.

### 4.4. Optic Cup Segmentation Results

**Figure 10:**
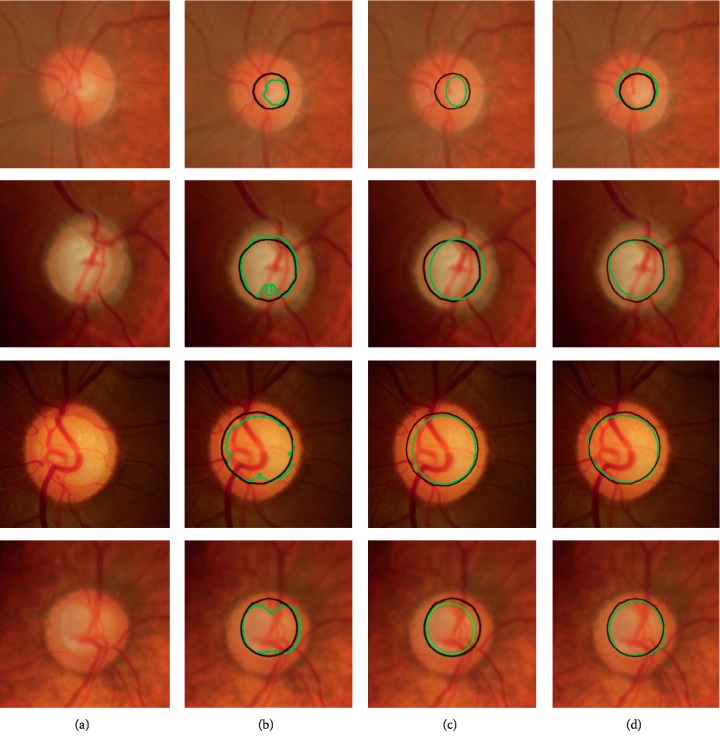
Optic cup segmentation results. (a) Original color image, (b) thresholding [24], (c) SWFCM clustering [42], and (d) LSACM-SP. Black line: ground truth and green line: detected results obtained by an approach.

For cup segmentation, we employ two different approaches, i.e., thresholding [24] and clustering [42], for comparison. The corresponding comparison results obtained from different approaches are shown in Figure 10.

According to Figure 10, it can be seen that our approach achieves small deviation of the detected optic cup boundary from the ground truth both on the nasal and temporal sides. However, other approaches suffer from a significant influence on the segmentation accuracy, especially for dense blood vessels presented on the nasal side. Specially, our approach is always superior to the nonjoint approaches [24, 42] due to the fact that our approach makes full use of the useful prior of optic disc boundary for optic cup boundary extraction. Comparing with the existing approaches, our approach has the following advantages: (1) intensity inhomogeneity, a frequently occurring phenomenon within optic cup is addressed, and thereby the discrimination between the optic cup and nonoptic cup is enhanced. (2) The proposed structure prior can guide the optic cup contour evolution in an effective region, which can reduce the negative effect of nonobjects, generating robust and reliable segmentation results. (3) Our approach is free from any training process and shape constraint, which is robust and effective in capturing a large range of intensity and shape variations. More detailed quantitative assessment results of the optic cup segmentation using DI and boundary-based distance criteria are shown in Table 3. As seen from Table 3, the proposed approach outperforms others comparison approaches in terms of the two important segmentation measurements.

Furthermore, the pairwise one-tailed *t*-tests [43] are used to verify the effectiveness of our approach for optic cup segmentation. The corresponding results are depicted in Table 4. Seen from Table 4, all the *p* values are much less than 0.05, indicating that the null hypotheses are disapproved in all pairwise tests. As a whole, the proposed approach shows a significant improvement in the optic cup segmentation results.

### 4.5. Glaucoma Assessment

**Figure 11:**
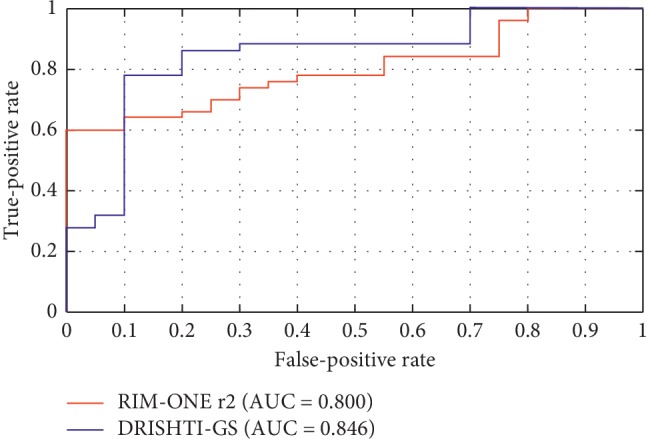
The ROC curves of LSACM-SP in glaucoma detection on the Drishti-GS and RIM-ONE r2 databases.

In this section, we will give the performance of the glaucoma detection based on our approach. Since the vertical CDR value is one of the most important indicators for glaucoma detection, we use the segmented optic disc and optic cup results to calculate the vertical CDR. Here, the normalized CDR value ${\hat{Q}}_{i}$ of the *i*th image can be calculated by

(17) $$\begin{array}{c} {\hat{Q}}_{i}=\frac{Q_{i}-Q_{\min}}{Q_{\max}-Q_{\min}}, \end{array}$$

where *Q*_max_ and *Q*_min_ are the maximum and minimum vertical CDR values. Here, the area under ROC curve (AUC) is used for glaucoma assessment, as shown in Figure 11.

### 4.6. Comparison with the State-of-the-Art Approaches

After the optic disc and optic cup boundaries extraction, we use the accuracy as a common measurement for glaucoma assessment. In this section, some state-of-the-art glaucoma diagnosis approaches are employed for verifying the effectiveness of the proposed approach, i.e., superpixel segmentation [3], wavelet features [44], multifeature fusion [45], deep learning [46], SDC [47], and AWLCSC [48].  Table 5 shows the classification results obtained by different algorithms on the DRISHTI-GS and RIM-ONE r2 databases. According to the comparison result, we can learn that the proposed LSACM-SP achieves a promising performance.

Besides, we also use the pairwise one-tailed *t*-tests on accuracy to further verify the effectiveness of the proposed approach. The results are shown in Table 6. From these results, we can learn that the *p* values obtained by the pairwise one-tailed *t*-tests are less than 0.05, which indicates that our approach significantly outperforms other glaucoma diagnosis approaches.

## 5. Conclusions

Since glaucomatous damage is irreversible, automated assessment of glaucoma is of interest in early detection and treatment. A novel model for simultaneous segmenting optic disc and optic cup for glaucoma diagnosis is presented in this paper. First, LSACM is introduced to overcome the influence caused by intensity inhomogeneity. Then, the proposed approach is presented by combining structure prior consisting of the hierarchical image segmentation and the attraction term. After that our approach is updated iteratively and constantly adjusted both the optic disc and optic cup boundaries to approximate the true object boundaries. The proposed approach is tested and evaluated on two publicly available DRISHTI-GS and RIM-ONE r2 databases. Seen from the experimental results, the proposed approach outperforms the state-of-the-art approaches. Although good segmentation performance can be achieved by the proposed approach, it may fail in some of the normal fundus images with extremely small optic cup sizes. In the future, we will introduce the priors on the location and shape of optic disc and optic cup to overcome this issue.

## Figures and Tables

**Table 1:** Optic disc segmentation results on the DRISHTI-GS and RIM-ONE r2 databases.

Methods	DI (DRISHTI-GS)	Boundary-based distance (DRISHTI-GS)	DI (RIM-ONE r2)	Boundary-based distance (RIM-ONE r2)
GVF [16]	0.867	39.239	0.735	42.768
C-V [24]	0.885	26.578	0.734	29.648
LIC [41]	0.910	11.900	0.779	15.764
Superpixel [3]	0.932	10.747	0.816	13.611
LSACM [29]	0.931	10.012	0.808	13.155
**LSACM-SP**	**0.955**	**8.711**	**0.853**	**10.232**

**Table 2:** The *p* values of the pairwise one-tailed *t*-tests of LSACM-SP and other optic disc segmentation approaches on DI.

Methods	*p* values (DRISHTI-GS)	Methods	*p* values (RIM-ONE r2)
LSACM-SP vs. GVF	3.43*e *−* *05	LSACM-SP vs. GVF	1.37*e *−* *06
LSACM-SP vs. C-V	3.44*e *−* *05	LSACM-SP vs. C-V	2.80*e *−* *05
LSACM-SP vs. LIC	1.35*e *−* *04	LSACM-SP vs. LIC	5.35*e *−* *05
LSACM-SP vs. superpixel	8.90*e *−* *04	LSACM-SP vs. superpixel	6.32*e *−* *04
LSACM-SP vs. LSACM	4.34*e *−* *03	LSACM-SP vs. LSACM	8.73*e *−* *04

**Table 3:** Optic cup segmentation results on the DRISHTI-GS and RIM-ONE r2 databases.

	DI (DRISHTI-GS)	Boundary-based distance (DRISHTI-GS)	DI (RIM-ONE r2)	Boundary-based distance (RIM-ONE r2)
Thresholding [24]	0.625	51.337	0.602	56.658
SWFCM clustering [42]	0.803	26.361	0.741	29.874
**LSACM-SP**	**0.847**	**20.863**	**0.785**	**23.245**

**Table 4:** The *p* values of the pairwise one-tailed *t*-tests of LSACM-SP and other optic cup segmentation approaches on DI.

Methods	*p* values (DRISHTI-GS)	Methods	*p* values (RIM-ONE r2)
LSACM-SP vs. Thresholding	8.12*e* − 04	LSACM-SP vs. Thresholding	1.26*e* − 05
LSACM-SP vs. SWFCM clustering	1.04*e* − 03	LSACM-SP vs. SWFCM clustering	2.21*e* − 03

**Table 5:** Comparison of the proposed approach against the state-of-the-art approaches on the DRISHTI-GS and RIM-ONE r2 databases.

Methods	Accuracy (%) DRISHTI-GS	Accuracy (%) RIM-ONE r2
Superpixel segmentation [3]	69.27	78.64
Wavelet features [44]	40.19	59.81
Multifeature fusion [45]	75.84	83.57
Deep learning [46]	84.38	80.18
SDC [47]	87.25	84.17
AWLCSC [48]	88.63	85.56
**LSACM-SP**	**89.01**	**84.69**

**Table 6:** The *p* values of the pairwise one-tailed *t*-tests of LSACM-SP and other glaucoma diagnosis approaches on accuracy.

Methods	*p* values (DRISHTI-GS)	Methods	*p* values (RIM-ONE r2)
LSACM-SP vs. superpixel	9.34*e* − 04	LSACM-SP vs. superpixel	5.56*e* − 04
LSACM-SP vs. wavelet features	5.98*e* − 05	LSACM-SP vs. wavelet features	2.92*e* − 05
LSACM-SP vs. multifeature fusion	0.0097	LSACM-SP vs. multifeature fusion	0.0014
LSACM-SP vs. deep learning	0.0110	LSACM-SP vs. deep learning	0.0019
LSACM-SP vs. SDC	0.0183	LSACM-SP vs. SDC	0.0272
LSACM-SP vs. AWLCSC	0.0421	LSACM-SP vs. AWLCSC	0.0303

## Data Availability

The databases used in our study are all available publicly, and these data used to support the findings of this study are included within the following articles. The DRISHTI-GS database is available in Sivaswamy et al. [38]. The RIM-ONE r2 database is available in Fumero et al. [39].

## Appendix

Minimizing the energy functional *E*^LSACM‐SP^ with respect to *c*_1_, *c*_2_, *c*_3_, *c*_4_, *B*_Disc_(**x**), *B*_Cup_(**x**), *σ*_1_, *σ*_2_, *σ*_3_, and *σ*_4_, we can achieve the minimizer for each variable. That is,

(1) *Energy minimization with respect to*{*c*_*i*_, *σ*_*i*_}, *B*_Disc_(**x**), *B*_Cup_(**x**): functionals for {*c*_*i*_, *σ*_*i*_}, *B*_Disc_(**x**), *B*_Cup_(**x**) of *E*^LSACM‐SP^ are givens as
  (A.1) $$\begin{array}{c} \varepsilon_{1}\left(B_{\text{Disc}}\left(x\right),c_{1},\sigma_{1}\right)=\int_{\Omega }\int_{\Omega }K_{\rho }\left(x,y\right)\left(\log\left(\sigma_{1}\right)+\left(\frac{{\left(I\left(y\right)-B_{\text{Disc}}\left(x\right)c_{1}\right)}^{2}}{2\sigma_{1}^{2}}\right)\right)H\left(\phi \left(y\right)\right)\text{d}x\text{ d}y,\\ \varepsilon_{2}\left(B_{\text{Disc}}\left(x\right),c_{2},\sigma_{2}\right)=\int_{\Omega }\int_{\Omega }K_{\rho }\left(x,y\right)\left(\log\left(\sigma_{2}\right)\right)+\left(\frac{{\left(I\left(y\right)-B_{\text{Disc}}\left(x\right)c_{2}\right)}^{2}}{2\sigma_{2}^{2}}\right)\left(1-H\left(\phi \left(y\right)\right)\right)\text{d}x\text{ d}y,\\ \varepsilon_{3}\left(B_{\text{Cup}}\left(x\right),c_{3},\sigma_{3}\right)=\int_{\Omega }\int_{\Omega }K_{\rho }\left(x,y\right)\left(\log\left(\sigma_{3}\right)+\left(\frac{{\left(I\left(y\right)-B_{\text{Cup}}\left(x\right)c_{3}\right)}^{2}}{2\sigma_{3}^{2}}\right)\right)H\left(\phi \left(y\right)\right)\left(H\left(\varphi \left(y\right)\right)\right)\text{d}x\text{ d}y,\\ \varepsilon_{4}\left(B_{\text{Cup}}\left(x\right),c_{4},\sigma_{4}\right)=\int_{\Omega }\int_{\Omega }K_{\rho }\left(x,y\right)\left(\log\left(\sigma_{4}\right)+\left(\frac{{\left(I\left(y\right)-B_{\text{Cup}}\left(x\right)c_{4}\right)}^{2}}{2\sigma_{4}^{2}}\right)\right)H\left(\phi \left(y\right)\right)\left(1-H\left(\varphi \left(y\right)\right)\right)\text{d}x\text{ d}y, \end{array}$$
Each *functional* in (A.1) has an explicit minimizer for each variable. That is,
  (A.2) $$\begin{array}{c} c_{1}=\frac{\int_{\Omega }\left[K_{\rho }\left(x,y\right)⊗B_{\text{Disc}}\left(x\right)\right]I\left(y\right)M_{\text{Disc\_}1}\text{d}y}{\int_{\Omega }\left[K_{\rho }\left(x,y\right)⊗B_{\text{Disc}}^{2}\left(x\right)\right]M_{\text{Disc\_}1}\text{d}y}, \end{array}$$
  (A.3) $$\begin{array}{c} c_{2}=\frac{\int_{\Omega }\left[K_{\rho }\left(x,y\right)⊗B_{\text{Disc}}\left(x\right)\right]I\left(y\right)M_{\text{Disc\_}2}\text{d}y}{\int_{\Omega }\left[K_{\rho }\left(x,y\right)⊗B_{\text{Disc}}^{2}\left(x\right)\right]M_{\text{Disc\_}2}\text{d}y}, \end{array}$$
  (A.4) $$\begin{array}{c} c_{3}=\frac{\int_{\Omega }\left[K_{\rho }\left(x,y\right)⊗B_{\text{Cup}}\left(x\right)\right]I\left(y\right)M_{\text{Cup\_}3}\text{d}y}{\int_{\Omega }\left[K_{\rho }\left(x,y\right)⊗B_{\text{Cup}}^{2}\left(x\right)\right]M_{\text{Cup\_}3}\text{d}y}, \end{array}$$
  (A.5) $$\begin{array}{c} c_{4}=\frac{\int_{\Omega }\left[K_{\rho }\left(x,y\right)⊗B_{\text{Cup}}\left(x\right)\right]I\left(y\right)M_{\text{Cup\_}4}\text{d}y}{\int_{\Omega }\left[K_{\rho }\left(x,y\right)⊗B_{\text{Cup}}^{2}\left(x\right)\right]M_{\text{Cup\_}4}\text{d}y}, \end{array}$$
  (A.6) $$\begin{array}{c} B_{\text{Disc}}\left(x\right)=\frac{K_{\rho }\left(x,y\right)⊗\left[I\left(y\right)M_{\text{Disc\_}1}\right]\left(c_{1}/\sigma_{1}^{2}\right)+K_{\rho }\left(x,y\right)⊗\left[I\left(y\right)M_{\text{Disc\_}2}\right]\left(c_{2}/\sigma_{2}^{2}\right)}{K_{\rho }\left(x,y\right)⊗M_{\text{Disc\_}1}\left(c_{1}^{2}/\sigma_{1}^{2}\right)+K_{\rho }\left(x,y\right)⊗M_{\text{Disc\_}2}\left(c_{2}^{2}/\sigma_{2}^{2}\right)}, \end{array}$$
  (A.7) $$\begin{array}{c} B_{\text{Cup}}\left(x\right)=\frac{K_{\rho }\left(x,y\right)⊗\left[I\left(y\right)M_{\text{Cup\_}3}\right]\left(c_{3}/\sigma_{3}^{2}\right)+K_{\rho }\left(x,y\right)⊗\left[I\left(y\right)M_{\text{Cup\_}4}\right]\left(c_{4}/\sigma_{4}^{2}\right)}{K_{\rho }\left(x,y\right)⊗M_{\text{Cup\_}3}\left(c_{3}^{2}/\sigma_{3}^{2}\right)+K_{\rho }\left(x,y\right)⊗M_{\text{Cup\_}4}\left(c_{4}^{2}/\sigma_{4}^{2}\right)}, \end{array}$$
  (A.8) $$\begin{array}{c} \sigma_{1}=\sqrt{\frac{\int_{\Omega }\int_{\Omega }K_{\rho }\left(x,y\right){\left(I\left(y\right)-B_{\text{Disc}}\left(x\right)c_{1}\right)}^{2}M_{\text{Disc}\_1}\text{d}x\text{ d}y}{\int_{\Omega }\int_{\Omega }K_{\rho }\left(x,y\right)M_{\text{Disc}\_1}\text{d}x\text{ d}y}}, \end{array}$$
  (A.9) $$\begin{array}{c} \sigma_{2}=\sqrt{\frac{\int_{\Omega }\int_{\Omega }K_{\rho }\left(x,y\right){\left(I\left(y\right)-B_{\text{Disc}}\left(x\right)c_{2}\right)}^{2}M_{\text{Disc}\_2}\text{d}x\text{ d}y}{\int_{\Omega }\int_{\Omega }K_{\rho }\left(x,y\right)M_{\text{Disc}\_2}\text{d}x\text{ d}y}}, \end{array}$$
  (A.10) $$\begin{array}{c} \sigma_{3}=\sqrt{\frac{\int_{\Omega }\int_{\Omega }K_{\rho }\left(x,y\right){\left(I\left(y\right)-B_{\text{Cup}}\left(x\right)c_{3}\right)}^{2}M_{\text{Cup}\_3}\text{d}x\text{ d}y}{\int_{\Omega }\int_{\Omega }K_{\rho }\left(x,y\right)M_{\text{Cup}\_3}\text{d}x\text{ d}y},} \end{array}$$
  (A.11) $$\begin{array}{c} \sigma_{4}=\sqrt{\frac{\int_{\Omega }\int_{\Omega }K_{\rho }\left(x,y\right){\left(I\left(y\right)-B_{\text{Cup}}\left(x\right)c_{4}\right)}^{2}M_{\text{Cup}\_4}\text{d}x\text{ d}y}{\int_{\Omega }\int_{\Omega }K_{\rho }\left(x,y\right)M_{\text{Cup}\_4}\text{d}x\text{ d}y}}, \end{array}$$
where ⊗ denotes the convolution operator.
(2) *Energy minimization with respect toϕ* and *φ*: minimizing *E*^LSACM‐SP^ with respect to *ϕ* and *φ*, it can be solved by the standard gradient descent method. After a series of calculations, the solution is obtained as follows:
  (A.12) $$\begin{array}{c} \frac{\partial \phi }{\partial t}=\lambda \text{ div}\left(\frac{\nabla \phi }{\sqrt{\phi_{x}^{2}+\phi_{y}^{2}}}\right)\delta \left(\phi \right)-\left\{\left(F_{1}-F_{2}+\alpha F_{4}\right)+\left(\alpha F_{3}-\alpha F_{4}\right)H\left(\varphi \right)-\nu H\left(\varphi \right)\right\}\delta \left(\phi \right), \end{array}$$
  (A.13) $$\begin{array}{c} \frac{\partial \varphi }{\partial t}=\mu \text{ div}\left(\frac{\nabla \varphi }{\sqrt{\varphi_{x}^{2}+\varphi_{y}^{2}}}\right)\delta \left(\varphi \right)-\left\{\left(\alpha F_{3}-\alpha F_{4}\right)H\left(\phi \right)+\nu \left(1-H\left(\phi \right)\right)\right\}\delta \left(\varphi \right), \end{array}$$
where ∇ denotes *the* gradient operator, div(·) is the divergence operator, and *δ*(*ϕ*) is the Dirac functional [29].
In this paper, a simple and stable approach [29] is employed to regularize the level set functions during each level set evolution. Therefore, level set functions depicted in (A.12) and (A.13) are regularized by the following formulas:
  (A.14) $$\begin{array}{c} \phi^{l+1}=\phi^{l}+\Delta t\cdot \nabla^{2}\phi^{l}, \end{array}$$
  (A.15) $$\begin{array}{c} \varphi^{l+1}=\varphi^{l}+\Delta t\cdot \nabla^{2}\varphi^{l}, \end{array}$$
where *ϕ*^*l*^*andφ*^*l*^ are two level set functions acquired at the *l*-th iteration, ∇^2^ is the Laplacian operator, and Δ*t* denotes time steps.
